# Over the Horizon: The Present and Future of Endovascular Neural Recording and Stimulation

**DOI:** 10.3389/fnins.2020.00432

**Published:** 2020-05-06

**Authors:** James Z. Fan, Victor Lopez-Rivera, Sunil A. Sheth

**Affiliations:** Department of Neurology, UTHealth McGovern Medical School, Houston, TX, United States

**Keywords:** electrode, endovascular approach, stentrode, iEEG (intracranial EEG), deep brain stimualtion

## Abstract

The past decade has witnessed an explosion in applications for neural recording and stimulation in the treatment of clinical disorders. Neuromodulatory approaches are now a mainstay of care for essential tremor and Parkinson’s disease, and are expanding rapidly into a wide range of other neurological and psychiatric diseases. In parallel, advancements in endovascular approaches to cerebrovascular diseases have resulted in minimally invasive techniques that deliver devices to neural tissue in the central and peripheral nervous systems, with significantly improved safety and efficacy. In this review, we discuss the history of endovascular neural recording and stimulation, its current progress, and applications for neurological disease.

## Existing Neural Recording and Modulatory Approaches for Neurological Disease

In recent years, there have been substantial improvements in methods to monitor and stimulate the brain’s electrical activity with a number of technologies developed. Intracranial electrical recording is being used with increasing frequency for diagnostic purposes in patients with epilepsy, in whom cortical grids and electrode strips are now complemented with high-density microelectrode arrays and stereotactic electroencephalogram (SEEG) leads. These techniques provide unparalleled spatial precision and resolution. Further, deep brain stimulation (DBS) has become a mainstay of therapy for movement disorders including motor symptoms of Parkinson’s disease and essential tremor (Volkmann and Deuschl, 2007; Kocabicak et al., 2015). As a non-invasive tool, transcranial magnetic stimulation (TMS) can deliver repetitive bursts of high-frequency waves to the brain cortex through the intact scalp for use in depression, migraine, and movement disorders (Daskalakis, 2014; Suppa et al., 2016; Berlim et al., 2017); however, lack of a standardized protocol for treatment of these disorders has led to variable results on its efficacy. Further, there has been a recent burst of enthusiasm in vagal nerve stimulation (VNS), which can be accomplished by a relatively minor surgical procedure and has received FDA clearance for the treatment of depression and migraines (Rush et al., 2000; Barbanti et al., 2015). Advantages and disadvantages of these technologies are detailed in Table 1.

**TABLE 1:** Neuromodulatory approaches including putative endovascular ones for treatment resistant major depressive disorder.

Neuromodulatory approach	Advantages	Disadvantages	Targets
DBS	-Permanent, continuous treatment -Precise and accurate targeting	Invasive implantation	Subgenual Cingulate Gyrus, Nucleus Accumbens, Anterior limb of Internal Capsule (Volkmann and Deuschl, 2007)
TMS	Non-invasive, short duration, and low-intensity stimulus	Variable efficacy and results, lack of a standardized protocol, treatment to be delivered by non-home-based equipment.	Multiple, commonly Dorsolateral Prefrontal cortex (Berlim et al., 2017)
VNS	FDA approved for treatment resistant depression	Incision needed to implant pulse generator. Unclear efficacy	Vagus nerve (Rush et al., 2000)
Endovascular Stimulation	Minimally invasive, access deep structures	-Antiplatelet Therapy	-Nucleus Accumbens (ACA, 2.2-2.6mm) (Aldaoud et al., 2018)
		-Limited Targeting	-Subgenual cingulate white matter (A2 ACA, 1.9-2.2mm) (Aldaoud et al., 2018)
		-Unclear long-term safety	-Vagus nerve (internal jugular vein)
			-Dorsolateral Prefrontal Cortex (middle meningeal artery)

*DBS, deep brain stimulation; TMS, transcranial magnetic stimulation; VNS, vagal nerve stimulation; ACA, anterior cerebral artery.*

## The Lure of Endovascular Delivery

Despite the successes detailed above, these diagnostic and treatment modalities all suffer tradeoffs between accuracy/efficacy and invasiveness. For example, while non-invasive TMS benefits from an ideal safety profile, its efficacy is limited due to the requirement for treatment to be delivered by non-home-based equipment, as well as the lack of direct contact of the power source with the target structure. There is also the possibility of off-target and non-specific effects. These limitations likely contribute to the observed relapse rates in patients with major depressive disorder (MDD) treated with TMS (Dannon et al., 2002). Conversely, invasive electroceutical approaches such as VNS and DBS physically engage with the target neural structure and are able to deliver continuous therapy. These devices are implanted chronically and minimize issues of compliance, which may contribute to their observed improved efficacy over time (Aaronson et al., 2017). On the other hand, the requirement for surgical implantation, even with relatively minimally invasive open surgical approaches, poses real and perceived risks. These risks have likely limited their incorporation more widely into clinical practice.

Endovascular approaches, however, address many of these limitations. Endovascular procedures are substantially less invasive than open surgical approaches, and the recovery time is minimal. Concerns for site infections are nearly absent. In addition, many high-value central and peripheral nervous system targets are adjacent to vascular structures. Delivery of an electroceutical device adjacent to these targets, through arterial or venous routes, may be a safer and more broadly appealing approach toward neuromodulation, particularly for disorders such as migraine and MDD, in which the procedural risk of any intervention would need to be low. Below, we detail some of the progress made in this field and discuss present challenges that will need to be addressed.

## Historical Development of Endovascular Recording

From small singular guidewires to stent-electrodes, endovascular monitoring and stimulation devices have evolved significantly, though several limitations and questions rose in their early development. In 1972, Penn et al. (1973) used a stainless-steel wire with a platinum cobalt magnet as an electroencephalogram (EEG) electrode placed in the carotid artery of baboons to monitor their brain activity. With the interceding skull and dural tissue no longer dampening the electrical signal, these electrodes were able to detect far greater amplitudes than scalp EEG. Proof-of-concept studies in humans began in the 1990s with a Seeker Lite-10 guide wire (0.31 mm in diameter with a platinum tip), used to improve detection of epileptic foci. Endovascular recordings from middle and anterior cerebral artery segments were taken from 14 patients without complication (Nakase et al., 1995). In three epilepsy patients, researchers concurrently implanted subdural electrodes and noted simultaneous spike discharges (Nakase et al., 1995). While these early experiments were promising, they suffered from a number of limitations, including a lack of understanding of these signals’ characteristics with respect to artifact from sources such as pulse, motion, and adjacent tissue. In addition, these recordings had very limited data capture as the recordings were brief, and spatial resolution was limited by constraints of arterial anatomy. As such, no independent conclusions could be drawn about the seizure foci enough to prove its clinical utility.

By the late 1990s, human experiments with similar platinum electrodes in the arterial system were shown to be capable of recording amplitudes evoked by somatosensory stimulus (Stoeter et al., 1995) and capable of detecting the disappearance of epileptiform discharges with valproic acid administration (García-Asensio et al., 1999). Consistent with prior studies, positioning of the electrode tip generated different signals, often with artifact, and made predicting the origin of signals difficult. One technique addressed this limitation by recording from the bilateral hemispheres, to attempt to subtract baseline artifact (García-Asensio et al., 1999). Further, in order to obtain endovascular recordings for longer periods of time, transvenous recordings were performed, as the safety profile of prolonged catheterization in the venous system is significantly better than for arteries. Successful recording with this approach was performed for periods of up to 75 h (Kunieda et al., 2000). However, due to its close proximity to the anterior and posterior mesial temporal lobe, the approach was severely limited by the need for the patient to remain still throughout the entire procedure, with any movement creating artifact. As such, persistent limitations of artifact, inconsistent signal recordings, limited duration of recording, limited spatial resolution and targeting, and uncertain safety profile in early approaches prevented widespread use of endovascular electrical recordings.

In the 21st century, devices have been addressing these challenges by focusing on miniaturization of electrodes, the creation of electrode arrays, and long-term implantable devices. Llinas et al experimented with such arrays to measure peripheral nerve activity by using four 20μm electrodes made from iron and platinum black wires (Llinás et al., 2005). *In vitro* endovascular recordings from the spinal cord also compared well to surface electrodes. In 2013, Bower et al. (2013) used the first venous catheter recording device made from 16 microelectrodes. Placing the microelectrodes in the superior sagittal sinus (SSS), microspikes were observed from microelectrodes that were missed both on subdural and intravascular macroelectrodes (Bower et al., 2013). Through the usage of smaller probes, electrodes retained a fidelity of up to 1 kHz, enabling recording of high frequency oscillations, improving applicability toward seizure localization. However, these experiments only recorded for relatively brief intervals, and spatial resolution challenges remained.

## Stentrode

The furthest developed endovascular electrical recording and stimulation technology to date is the Stentrode (Synchron, Inc). By delivering an expandable electrode array with at least seven electrodes, the Stentrode aimed to address the shortcomings of prior techniques. Placement of multiple electrodes was theorized to provide better spatial resolution and its self-expanding property, fixating the electrodes on the vessel wall, aimed to delivery consistent signal during chronic implantation. In 2016, Oxley et al. (2016) studied the placement of endovascular electrodes during a 190-day period. Stentrodes were placed in ovine SSS adjacent to sensorimotor cortex. Endothelialization and wall incorporation of the Stentrode occurred six days after implantation, and the electrodes demonstrated a stable maximum recording bandwidth throughout the implantation period. During this time, the Stentrode had a similar power spectrum and recording bandwidth compared to epidural arrays, but slightly inferior to subdural arrays (Oxley et al., 2016).

A subsequent study explored Stentrode’s spatial resolution. Both spatial resolution and signal to noise ratio from endovascular arrays were comparable to those of subdural and epidural arrays three weeks after implantation (John et al., 2018). However, spatial resolution depended on array location relative to targets as well as recording frequency; as such, because delivery of the device is constrained by the location and course of blood vessels, the absence of a suitable vessel adjacent to a target structure would remain a limitation. The device is currently being tested as a brain-machine interface for thought-controlled guidance of limb movement in patients with paralysis.

Although promising, endovascular recording continues to suffer from specific challenges that limit clinical application. Signal quality, and artifacts from thermal noise, movement and ambient/off-target electrical signals remain limitations. Progress in this area, however, has been rapid in the last 5 years. Whereas the literature on cerebral endovascular electrical recording and progress in the field was relatively limited in the early part of this century, there have been multiple efforts from numerous groups in the last several years focused on improving recording and decoding accuracy (He et al., 2016; John et al., 2018).

## Development of Endovascular Stimulation and Perspectives for the Future

While the majority of prior work on endovascular approaches have been on recording, with advances in device design the possibility of clinically applicable endovascular stimulators continues to improve. Initial efforts have examined existing targets of surgically implanted lead-based DBS. A computational model by Teplitzky et al. (2014) examined 17 targets of DBS and reported that 5 of these targets including the fornix and subgenual cingulate white matter tracts were adjacent to vascular structures that could be realistically catheterized. In 2018, Opie et al. (2018) tested Stentrode-based stimulation in the SSS near the primary motor cortex of sheep. Stentrode activation caused lip, face, jaw, neck, and limb movements in electrodes anterior to the cruciate sulcal veins, a previously noted landmark for the sheep motor cortex (He et al., 2016). In order for this field to advance, however, key questions remain in choosing the optimal target for stimulation/disease-state, as well as design constraints for the endovascular device.

The ideal initial disease target would be one that is already known to benefit from neuromodulatory electrical stimulation, but for which existing treatments are felt to be too invasive, or for which a less invasive approach would substantially broaden the appeal of a neuromodulatory intervention. It should also be one for which long-term treatment would be expected. While movement disorder applications are the best studied conditions for stereotactic DBS, these diseases may not be ideal candidates for endovascular DBS, as vascular conduits to the commonly used tissue targets such as the subthalamic nucleus are suboptimal, and the stimulation amplitudes required to modulate these areas would likely lead to substantial off-target side-effects. Further, the spatial sub-millimeter targeting accuracy boasted by stereotactic placements will never be achievable with endovascular placement. On the other hand, neuro-psychiatric diseases including treatment-resistant MDD may benefit from endovascular approaches. Major depressive disorder has a high prevalence and over 50% of patients may be medication treatment resistant (Substance Abuse and Mental Health Services Administration, 2017). While neuromodulatory approaches have shown effect, surgical approaches such as VNS and DBS have had limited use, due in large part to their perceived invasiveness. While endovascular approaches may also require an incision, the size is relatively smaller, and performed in a less invasive manner, as the risk for postoperative cervical hematoma, vocal cord palsy, facial palsy, and hypoglossal nerve injury still need to be avoided (Kahlow and Olivecrona, 2013). Consequently, an endovascular stimulator that could be deployed through femoral access and placed adjacent to established MDD targets would be substantially less invasive and a potentially more appealing treatment option (Table 1).

Existing endovascular technology, however, will need to improve in several regards before these devices can be deployed. One key question is how these devices will be powered, and whether they will require wired connections to a battery pack, similar to stereotactic DBS. Such a setup, however, limits the ability to deploy them within arterial structures, as the likelihood of vessel injury and thrombosis is prohibitively great. To that end, a major advancement in endovascular electrical stimulators will be the development of wireless power delivery and control. Prior experiments using wired power delivery systems with tunneled lines through the jugular vein have demonstrated wire fatigue due to repetitive neck movements (Oxley et al., 2016). A wired pathway also provides a route for infection and if used for electrical recording, cause substantial interference (He et al., 2016). Aldaoud et al. (2018) studied inductive and capacitive coupling to transit power to a stent device implanted within muscle tissue of up to 30 mm depth. These methods of near-field power wireless power transfer achieved efficiencies of up to 2.6%. Deeper implantation, however, significantly limited transfer efficiency. Magnetoelectric (ME) materials provide another alternative. Here, a voltage is generated by mechanical coupling between magnetostrictive and piezoelectric layers in a thin film (Wickens et al., 2018). The benefit compared to induction is that a significantly smaller implant can be used that requires much weaker magnetic fields. Wickens et al. (2018) used ME stimulators placed the subthalamic nucleus to apply a biphasic 200 Hz stimulus and noted significant changes in behavior of hemi-parkinsonian rats. Rice-sized ME stimulators were developed for a human model with a corresponding magnetic stimulation coil being worn around the head (Wickens et al., 2018). Future miniaturized ME stimulators may be more flexible and navigable, enabling deployment within blood vessels.

Other design constraints include continued miniaturization, and improved deliverability. These devices would most likely be permanent implants; a retrievable option would be advantageous particularly as patients would likely be required to take anti-thrombotic medication while the devices are in place. Magnetic Resonance Imaging (MRI) compatibility will be crucial. Finally, long-term safety remains unknown. Only recently have experiments tested the long-term implantation of endovascular electrical devices. In animal models, devices such as the Stentrode have successfully remained patent with aspirin for up to 190 days (Oxley et al., 2016). However, antiplatelet therapy adds additional potential risks, as the optimal duration and antiplatelet regimen remains unknown. Long-term antiplatelet therapy is known to carry an increased risk of systemic hemorrhage and may be required to prevent in-stent or stent-adjacent thrombosis while allowing stent endothelization (Saber et al., 2018). For example, in the setting of venous sinus stenting, 3-month antiplatelet therapy is considered sufficient to prevent stent induced thrombosis, but practice remains variable and some interventionists have a preference toward longer periods (e.g., 4–6 months) (Nicholson et al., 2018). In addition, the effect of chronic endovascular electrical stimulation on vascular integrity remains uncharacterized, and may result in aneurysm formation or occlusion. Histological evaluation of blood vessel endothelium will be needed to understand whether long-term stimulation can be tolerated. At present, human safety and feasibility studies using the Stentrode are underway, with the first patient successfully implanted in September 2019 (Clinical Trials.gov, NCT03834857).

## Conclusion

Endovascular delivery of electrical recording and stimulation devices has the potential to improve upon the current neurosurgical procedures, with minimally invasive techniques that lower actual and perceived risks. At present, several limitations particularly safety, power, and long-term durability remain incompletely addressed; ongoing and future research efforts continue to address these challenges.

## Author Contributions

JF wrote first drafts of the manuscript. VL-R edited subsequent drafts and revisions. SS contributed to the conception and review of article. All authors contributed to manuscript revision, read and approved the submitted version.

## Conflict of Interest

SS reports a provisional patent covering endovascular electrical recording technology not discussed in this manuscript. The remaining authors declare that the research was conducted in the absence of any commercial or financial relationships that could be construed as a potential conflict of interest.
